# Supplementary material for: Smooth Interpolating Curves with Local Control and Monotone Alternating Curvature
Source: Comput Graph Forum. 2022 Oct 6;41(5):25–38. doi: 10.1111/cgf.14600 (PMC9827861; doi:10.1111/cgf.14600)
Supplement: Supplementary file 1 — Supplement Material [file CGF-41-25-s001.zip › Local-Smooth-Interpolating-MonoCurvature/extern/libhedra/coding-guidelines.html]

libigl


# Libigl Coding Tips (aka “How to code a SIGGRAPH project”)

This is a short list of coding tips that will greatly reduce your pain and suffering before (and after) the SIGGRAPH deadline.

### 1. Serialize it all

The entire state of your application should be serializable, i.e. It should be possible to save it into a binary file and reload it at any point. This drastically simplifies debugging, since you can serialize just before a crash happens and debug from that point without running your complete algorithm again. Serializing all results shown in the paper’s figures enables quicker editing iterations before (and after) the deadline. It also allows you to share your results with others that wish to compare with your method. An additional tip is to serialize the state of the application on the window close event and automatically reload it when you launch it again.

### 2. Always assert

Even if you know what you are doing, always assert, you will be surprised. Assertion is a powerful but underused feature available in all programming languages. It is essential for writing research code since often you will have to implement algorithms that turns out to not be doing what you expect: in these cases it is important to know if the algorithm is flawed or if there is a bug in your implementation. Discarding a good idea because of a coding bug is frustrating and unfortunately common. Assertion is an ideal way to reduce the chances of introducing bugs in your code and, differently from unit testing, requires a very minor programming effort. You should use them extensively.

### 3. Plot everything

If you can visually plot the results or some intermediate steps of your algorithm, do it, even if you think your implementation is correct! It is a lot easier to find bugs or to get an intuition on an algorithm by looking at a plot than by looking at the code.

### 4. If the compilation time after a code change is more than five seconds, you are doing it wrong

You will change your code hundreds of times every day for months. Let’s say that you will change it a hundred times a day (which is a very conservative estimate): if the compilation takes one minute, you will waste almost two hours every day, just waiting! What is even worse, is that since it is only 1–2 minutes at a time, it will not even be sufficient to prepare a coffee. Spend the hour or two that is needed to get your code to compile in a few seconds, you will benefit from it in the same day already, and the time saved over an entire project will be gigantic.

### 5. Commit often (and with a meaningful description)

Use a distributed version control system (git,hg), and keep the repository on a remote host. Commit often and put meaningful comments. This will serve you as an emergency backup and it will always allow you to have a running version of your code whenever your advisor is passing by and asking to see some results. She will be impressed and you will not have to quickly fix your build with your boss breathing down your neck.

### 6. Dependencies are evil, avoid them

Keep your code simple and with minimal external dependencies. Spending a day or two to code something from scratch while avoiding to use third party code is usually an investment that pays off. The more code you have in your algorithm that is not written by you, the harder debugging becomes. In particular, refrain from building your entire project on code that you do not understand to avoid bad surprises just before the deadline. If you must use code written by others, spend the time that is needed to fully understand what it does, and link it statically so that it will be easy to place breakpoints inside it.

### 7. Global variables are not evil, use them

Global variables are often extremely useful — if you think you need one, use it. They are indeed dangerous for large projects, but you are not coding one of those, you are coding a prototype to test a research idea. I suggest to keep one single copy of your entire application state in a global variable (or a singleton class) that can be serialized (see tip 1). This variable should include everything rendered on screen and all the temporary data produced by your algorithm. This will allow you to easily access all the data in your project for plotting or debugging purposes.

### 8. Prototype first

Don’t preemptively optimize and try to quickly write code that is clean and correct. It is common to try multiple different approaches to solve a new problem before finding the right one. This means that the majority of the code that you will write will not be used at the end of the project. While you should still write high-quality and bug-free code to make sure that your results is correct, you definitely do not want to spend time optimizing it before you are sure that is the right approach. In particular, it is helpful to learn a good prototyping language (Python, matlab) and use it for the early stages of the project and switch to (or mix it with) c++ only after finding a promising direction.

### 9. Avoid explicit pointers

Do yourself a favor, do not use explicit pointers. If you use a language that supports explicit pointers, use them only if you really have to. And even in that case, keep them isolated in a single file and be very careful with them. Writing data inside another variable by accident might not trigger a crash, and simply produce strange artifacts that might convince you that a promising research direction does not work, while the problem lies in a nasty bug in your code. There is no reason to take that risk during prototyping, just avoid them and leave them for the end of the project in case they become necessary to optimize your code.

### 10. If your program crashes, fix it now!

If your program crashes, don’t close your eyes and move on. Try to make it happen again, debug it and fix it immediately. These bugs are a nightmare to find, and the more code you add on top of a bug will just make it harder to find. If you don’t fix it, due to Murphy’s law, it will start to be problematic only a few days before the deadline and you will have no time to fix it at that point.

*Daniele Panozzo*
